# Giant superconducting fluctuations in the compensated semimetal FeSe at the BCS–BEC crossover

**DOI:** 10.1038/ncomms12843

**Published:** 2016-09-30

**Authors:** S. Kasahara, T. Yamashita, A. Shi, R. Kobayashi, Y. Shimoyama, T. Watashige, K. Ishida, T. Terashima, T. Wolf, F. Hardy, C. Meingast, H. v. Löhneysen, A. Levchenko, T. Shibauchi, Y. Matsuda

**Affiliations:** 1 Department of Physics, Kyoto University, Kyoto 606-8502, Japan; 2 Research Center for Low Temperature and Materials Sciences, Kyoto University, Kyoto 606-8501, Japan; 3 Institute of Solid State Physics, Karlsruhe Institute of Technology, Karlsruhe D-76021, Germany; 4 Department of Physics, University of Wisconsin-Madison, Madison, Wisconsin 53706, USA; 5 Department of Advanced Materials Science, University of Tokyo, Kashiwa, Chiba 277-8561, Japan

## Abstract

The physics of the crossover between weak-coupling Bardeen–Cooper–Schrieffer (BCS) and strong-coupling Bose–Einstein condensate (BEC) limits gives a unified framework of quantum-bound (superfluid) states of interacting fermions. This crossover has been studied in the ultracold atomic systems, but is extremely difficult to be realized for electrons in solids. Recently, the superconducting semimetal FeSe with a transition temperature *T*_c_=8.5 K has been found to be deep inside the BCS–BEC crossover regime. Here we report experimental signatures of preformed Cooper pairing in FeSe, whose energy scale is comparable to the Fermi energies. In stark contrast to usual superconductors, large non-linear diamagnetism by far exceeding the standard Gaussian superconducting fluctuations is observed below T*∼20 K, providing thermodynamic evidence for prevailing phase fluctuations of superconductivity. Nuclear magnetic resonance and transport data give evidence of pseudogap formation at ∼T*. The multiband superconductivity along with electron–hole compensation in FeSe may highlight a novel aspect of the BCS–BEC crossover physics.

## Abstract (web summary)

The crossover between the weak-coupling limit and strong-coupling limit provides important information for quantum bound states of interacting fermions. Here, Kasahara *et al*. report thermodynamic evidence for prevailing phase fluctuations of superconductivity, highlighting unusual normal state in the BCS-BEC crossover regime.

## Graphical abstract

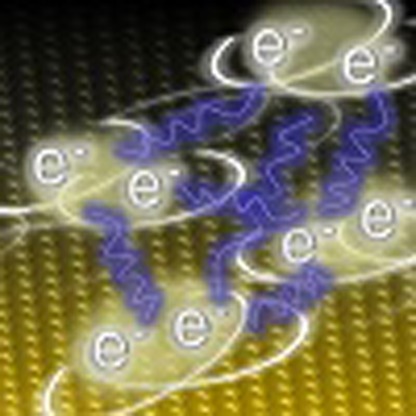

In the Bardeen–Cooper–Schrieffer (BCS) regime, weakly coupled pairs of fermions form the condensate wave function, while in the Bose–Einstein condensate (BEC) regime, the attraction is so strong that the fermions form local molecular pairs with bosonic character. The physics of the crossover is described by two length scales, the average pair size or coherence length *ξ*_pair_ and the average interparticle distance 1/*k*_F_, where *k*_F_ is the Fermi wave number. In the BCS regime, the pair size is very large and *k*_F_*ξ*_pair_≫1, while local molecular pairs in the BEC regime lead to *k*_F_*ξ*_pair_≪1. The crossover regime is characterized by *k*_F_*ξ*_pair_∼1, or equivalently the ratio of superconducting gap to Fermi energy Δ/*ɛ*_F_ of the order of unity. In this crossover regime, the pairs interact most strongly and new states of interacting fermions may appear; preformed Cooper pairing at much higher temperature than *T*_c_ is theoretically proposed^1^,^2^. Experimentally, however, such preformed pairing associated with the BCS–BEC crossover has been controversially debated in ultracold atoms^3^,^4^ and cuprate superconductors^5^,^6^,^7^,^8^. Of particular interest is the pseudogap formation associated with the preformed pairs that lead to a suppression of low-energy single-particle excitations. Also important is the breakdown of Landau's Fermi liquid theory due to the strong interaction between fermions and fluctuating bosons. In ultracold atomic systems, this crossover has been realized by tuning the strength of the interparticle interaction via the Feshbach resonance. In these artificial systems, Fermi liquid-like behaviour has been reported in thermodynamics even in the middle of crossover^3^, but more recent photoemission experiments have suggested a sizeable pseudogap opening and a breakdown of the Fermi liquid description^4^.

On the other hand, for electron systems in bulk condensed matter, it has been extremely difficult to access the crossover regime. Perhaps, the most frequently studied systems have been underdoped high-*T*_c_ cuprate superconductors^5^,^6^,^7^,^8^ with substantially shorter coherence length than conventional superconductors. In underdoped cuprates, pseudogap formation and non-Fermi liquid behaviour are well established, and unusual superconducting fluctuations have also been found above *T*_c_ (refs 6, 7). However, the pseudogap appears at a much higher temperature than the onset temperature of superconducting fluctuations^8^. It is still unclear whether the system is deep inside the crossover regime and to what extent the crossover physics is relevant to the phase diagram in underdoped cuprates. It has been also suggested that in iron-pnictide BaFe_2_(As_1−*x*_P_*x*_)_2_, the system may approach the crossover regime in the very vicinity of a quantum critical point^9^,^10^, but the fine-tuning of the material to a quantum critical point by chemical substitution is hard to accomplish. Therefore, this situation calls for a search of new systems in the crossover regime.

Among different families of iron-based superconductors, iron chalcogenides FeSe_*x*_Te_1−*x*_ exhibit the strongest band renormalization due to electron correlations, and recent angle-resolved photoemission spectroscopy studies for *x*=0.35−0.4 have shown that some of the bands near the Brillouin zone centre have very small Fermi energy, implying that the superconducting electrons in these bands are in the crossover regime^11^,^12^. Among the members of the iron chalcogenide series, FeSe (*x*=0) with the simple crystal structure formed of tetrahedrally bonded layers of iron and selenium is particularly intriguing. FeSe undergoes a tetragonal–orthorhombic structural transition at *T*_s_≈90 K, but in contrast to other Fe-based superconductors, no long-range magnetic ordering occurs at any temperature. Recently, the availability of high-quality bulk single crystals grown by chemical vapour transport^13^ has reopened investigations into the electronic properties of FeSe. Several experiments performed on these crystals have shown that all Fermi surface bands are very shallow^14^,^15^,^16^; one or two electron pockets centred at the Brillouin zone corner with Fermi energy 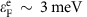, and a compensating cylindrical hole pocket near the zone centre with 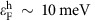. FeSe is a multigap superconductor with two distinct superconducting gaps Δ_1_≈3.5 and Δ_2_≈2.5 meV (ref. 14). Remarkably, the Fermi energies are comparable to the superconducting gaps; Δ/*ɛ*_F_ is ∼0.3 and ∼1 for hole and electron bands, respectively^14^. These large Δ/*ɛ*_F_(≈1/(*k*_F_*ξ*_pair_)) values indicate that FeSe is in the BCS–BEC crossover regime. In fact, values of 2Δ_1_/*k*_B_*T*_c_≈9 and 2Δ_2_/*k*_B_*T*_c_≈6.5, which are significantly enhanced with respect to the weak-coupling BCS value of 3.5, imply that the attractive interaction holding together the superconducting electron pairs takes on an extremely strong-coupling nature, as expected in the crossover regime. Moreover, the appearance of a new high-field superconducting phase when the Zeeman energy is comparable to the gap and Fermi energies, *μ*_0_*H*∼Δ∼*ɛ*_F_, suggests a peculiar superconducting state of FeSe (ref. 14). Therefore, FeSe provides a new platform to study the electronic properties in the crossover regime.

Here we report experimental signatures of preformed Cooper pairing in FeSe below T*∼20 K. Our highly sensitive magnetometry, thermoelectric and nuclear magnetic resonance (NMR) measurements reveal an almost unprecedented giant diamagnetic response as a precursor to superconductivity and pseudogap formation below T*. This yields profound implications on exotic bound states of strongly interacting fermions. Furthermore, the peculiar electronic structure with the electron–hole compensation in FeSe provides a new playground to study unexplored physics of quantum-bound states of interacting fermions.

## Results

### Giant superconducting fluctuations

It is well known that thermally fluctuating droplets of Cooper pairs can survive above *T*_c_. These fluctuations arise from amplitude fluctuations of the superconducting order parameter and have been investigated for many decades. Their effect on thermodynamic, transport and thermoelectric quantities in most superconductors is well understood in terms of standard Gaussian fluctuation theories^17^. However, in the presence of preformed pairs associated with the BCS–BEC crossover, superconducting fluctuations are expected to be strikingly enhanced compared with Gaussian theories due to additional phase fluctuations. Moreover, it has been suggested that such enhanced fluctuations can lead to a reduction of the density of states (DOS), dubbed the pseudogap^1^,^2^.

**Figure 1:**
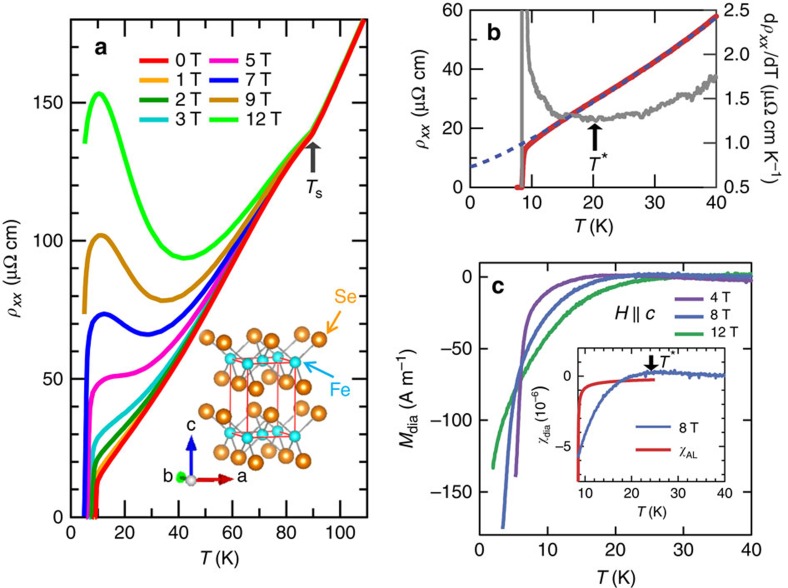
Excess conductivity and diamagnetic response of a high-quality single crystal of FeSe. (**a**) *T* dependence of *ρ*_*xx*_ in magnetic fields (**H**||*c*). The structural transition occurs at *T*_s_=90 K, which is accompanied by a kink in *ρ*_*xx*_(*T*). Inset shows the crystal structure of FeSe. (**b**) *T* dependence of *ρ*_*xx*_ (red) and d*ρ*_*xx*_/d*T* (grey). Below T* shown by arrow, *ρ*_*xx*_ shows a downward curvature. The blue dashed line represents *ρ*_*xx*_(*T*)=*ρ*_0_+*AT*^*α*^ with *ρ*_0_=7 μΩ cm *A*=0.6 μΩ cm K^−2^ and *α*=1.2. (**c**) Diamagnetic response in magnetization *M*_dia_ for **H**||*c*. The inset shows the diamagnetic susceptibility *χ*_dia_ at 8 T (blue) compared with the estimated *χ*_AL_ in the standard Gaussian fluctuations theory (red).

Quite generally, superconducting fluctuations give rise to an enhancement of the normal-state conductivity, which manifests itself as a downturn towards lower *T* of the resistivity versus temperature curve above *T*_c_. The high-field magnetoresistance of compensated semimetals is essentially determined by the product of the scattering times of electron and hole bands^14^. The large, insulating-like upturn in *ρ*_*xx*_(*T*) at high fields is thus an indication of the high quality of our crystals (Fig. 1a). At low temperatures, however, the expected downturn behaviour is observed, implying large superconducting fluctuations. Even at zero field, d*ρ*_*xx*_(*T*)/d*T* shows a minimum around T*∼20 K (Fig. 1b), indicating the appearance of excess conductivity below ∼T*. However, a quantitative analysis of this excess conductivity is difficult to achieve because it strongly depends on the extrapolation of the normal-state resistivity above T* to lower *T*. In addition, the resistivity may be affected by a change of the scattering time when a pseudogap opens at T* as observed in underdoped cuprates^18^.

We therefore examine the superconducting fluctuations in FeSe through the diamagnetic response in the magnetization. The magnetization *M*(*T*) for magnetic field **H** parallel to the *c* axis (Supplementary Fig. 1) exhibits a downward curvature below ∼T*. This pronounced decrease of *M*(*T*) can be attributed to the diamagnetic response due to superconducting fluctuations. Figure 1c shows the diamagnetic response in the magnetization *M*_dia_ between 0 and 40 K for *μ*_0_*H*=4, 8 and 12 T, obtained by subtracting a constant *M* as determined at 30 K. Although there is some ambiguity due to weakly temperature-dependent normal-state susceptibility, we find a rough crossing point in *M*_dia_(*T*, *H*) near *T*_c_. Such a crossing behaviour is considered as a typical signature of large fluctuations and has been found in cuprates^19^. The thermodynamic quantities do not include the Maki—Thompson-type fluctuations. Hence, the fluctuation-induced diamagnetic susceptibility of most superconductors including multiband systems can be well described by the standard Gaussian-type (Aslamasov–Larkin, AL) fluctuation susceptibility *χ*_AL_ (refs 20, 21, 22), which is given by


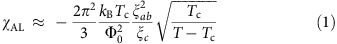


in the zero-field limit^23^. Here Φ_0_ is the flux quantum and *ξ*_*ab*_ (*ξ*_*c*_) is the effective coherence lengths parallel (perpendicular) to the *ab* plane at zero temperature. In the multiband case, the behaviour of *χ*_AL_ is determined by the shortest coherence length of the main band, which governs the orbital upper critical field. The diamagnetic contribution *χ*_AL_ is expected to become smaller in magnitude at higher fields, and thus |*χ*_AL_| yields an upper bound for the standard Gaussian-type amplitude fluctuations. In the inset of Fig. 1c, we compare *χ*_dia_ at 8 T with *χ*_AL_, where we use *ξ*_*ab*_ (=5.5 nm) and *ξ*_*c*_ (=1.5 nm)^14^,^15^. Obviously the amplitude of *χ*_dia_ of FeSe is much larger than that expected in the standard theory, implying that the superconducting fluctuations in FeSe are distinctly different from those in conventional superconductors.

The highly unusual nature of superconducting fluctuations in FeSe can also be seen in the low-field diamagnetic response. Since the low-field magnetization below 2 T is not reliably obtained from conventional magnetization measurements, we resort to sensitive torque magnetometry. The magnetic torque *τ*=*μ*_0_*V***M** × **H** is a thermodynamic quantity that has a high sensitivity for detecting magnetic anisotropy. Here *V* is the sample volume, **M** is the induced magnetization and **H** is the external magnetic field. For our purposes, the most important advantage of this method is that an isotropic Curie contribution from impurity spins is cancelled out^24^.

**Figure 2:**
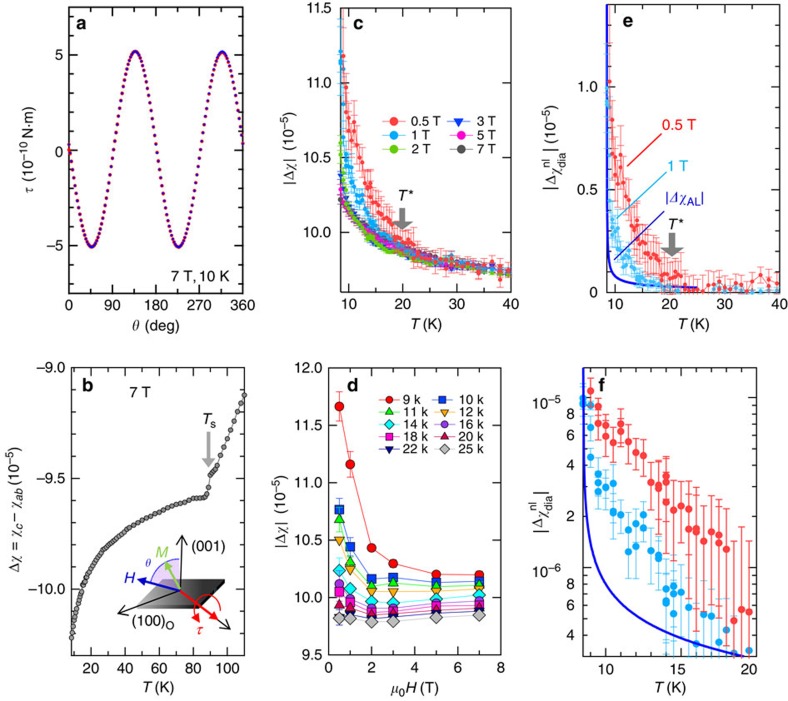
Diamagnetic response detected by magnetic torque measurements above *T*_c_. (**a**) The magnetic torque *τ* as a function of *θ*. Torque curves measured by rotating **H** in clockwise (red) and anticlockwise (blue) directions coincide (the hysteresis component is <0.01% of the total torque). (**b**) Anisotropy of the susceptibility between the *c* axis and *ab* plane, Δ*χ*, at 7 T. The inset is schematics of the *θ*-scan measurements. (**c**) The *T* dependence of |Δ*χ*| at various magnetic fields. (**d**) The *H* dependence of |Δ*χ*| at fixed temperatures. (**e**) Temperature dependence of the non-linear diamagnetic response at *μ*_0_*H*=0.5 T (red) and 1 T (blue) obtained by 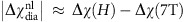. Blue line represents the estimated |Δ*χ*_AL_| in the standard Gaussian fluctuations theory. (**f**) 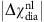 plotted in a semi-log scale at low temperatures. Error bars represent s.d. of the sinusoidal fit to the *τ*(*θ*) curves.

At each temperature and field, the angle-dependent torque curve *τ*(*θ*) is measured in **H** rotating within the *ac* (*bc*) plane, where *θ* is the polar angle from the *c* axis. In this geometry, the difference between the *c* axis and *ab* plane susceptibilities, Δ*χ*=*χ*_*c*_−*χ*_*ab*_, yields a *π*-periodic oscillation term with respect to *θ* rotation, 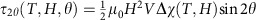 (Fig. 2a; Supplementary Fig. 2; Supplementary Note 1)^25^,^26^. In the whole measurement range, Δ*χ* is negative, that is, *χ*_*ab*_>*χ*_*c*_, which is consistent with magnetic susceptibility^27^ and NMR Knight-shift measurements^28^,^29^. Figure 2b shows the *T* dependence of Δ*χ* at 7 T, which is determined by the amplitude of the sinusoidal curve. At *T*_s_, Δ*χ*(*T*) exhibits a clear anomaly associated with the tetragonal–orthorhombic structural transition. On approaching *T*_c_, Δ*χ* shows a diverging behaviour. Figure 2c,d depicts the *T* and *H* dependence of |Δ*χ*|(*T*,*H*), respectively. Above T*∼20 K, |Δ*χ*|(*T*, *H*) is nearly field independent. Below T*, however, |Δ*χ*|(*T*,*H*) increases with decreasing *H*, indicating non-linear *H* dependence of *M*. This non-linearity increases steeply with decreasing temperature. Since |Δ*χ*| points to a diverging behaviour in the zero-field limit on approaching *T*_c_ (Fig. 2d), this strongly non-linear behaviour is clearly caused by superconducting fluctuations.

Thus, the diamagnetic response of FeSe contains *H*-linear and non-linear contributions to the magnetization; Δ*χ*(*T*) can be written as 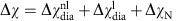, where 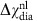 and 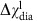 represent the diamagnetic contributions from non-linear and linear field dependence of magnetization, respectively, and Δ*χ*_N_ is the anisotropic part of the normal-state susceptibility, which is independent of *H*. Since Δ*χ*(*T*) is almost *H* independent at high fields (Fig. 2d), 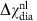 is estimated by subtracting *H*-independent terms from Δ*χ*. In Fig. 2e, we plot 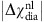 estimated from 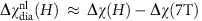, which we compare with the expectation from the Gaussian fluctuation theory at zero field given by 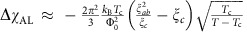. Near *T*_c_, 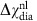 at 0.5 T is nearly 10 times larger than Δ*χ*_AL_. It should be noted that since 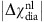 increases with decreasing *H*, 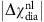 in the zero-field limit should be much larger than 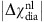 at 0.5 T. Thus, the non-linear diamagnetic response dominates the superconducting fluctuations when approaching *T*_c_ in the zero-field limit. We note that, although the AL diamagnetic contribution contains a non-linear term visible at low fields, this term is always smaller than the AL fluctuation contribution at zero field^20^,^21^,^22^.

Our magnetization and torque results provide thermodynamic evidence of giant superconducting fluctuations in the normal state of FeSe by far exceeding the Gaussian fluctuations. We stress that, since the energy scale of *k*_B_T*∼2 meV is comparable to 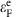, it is natural to attribute the observed fluctuations to preformed pairs associated with the BCS–BEC crossover. In the presence of those pairs, superconducting phase fluctuations^5^ arising from the mode coupling of fluctuations are expected to be significantly enhanced and to produce a highly non-linear diamagnetic response, as observed in the experiments. This non-linear response with large amplitude is profoundly different from the Gaussian behaviour in conventional superconductors.

### Pseudogap formation

**Figure 3:**
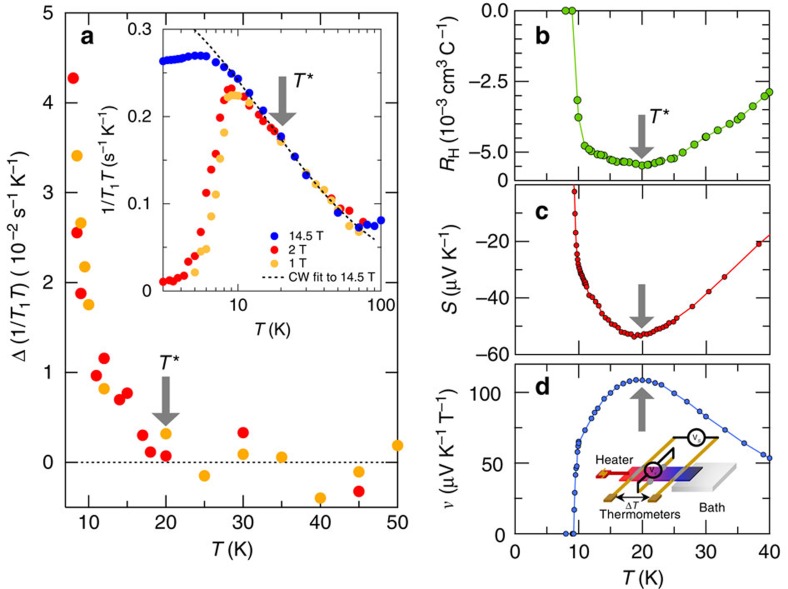
Possible pseudogap formation below T* evidenced by NMR and transport measurements. (**a**) Temperature dependence of the NMR relaxation rate divided by temperature 1/*T*_1_*T*. Inset: at 14.5 T, the temperature dependence of 1/*T*_1_*T* between ∼10 and 70 K is fitted to a Curie–Weiss law ∝(*T*+16 K)^−1^ (dashed line). Main panel: the difference between the Curie–Weiss fit and the low-field data Δ(1/*T*_1_*T*) is plotted as a function of temperature. (**b**) Hall coefficient, *R*_H_. (**c**) Seebeck coefficient, *S*. (**d**) Nernst coefficient, *ν*, in the zero-field limit as functions of temperature. Inset in **d** is a schematic of the measurement set-up of the thermoelectric coefficients.

Next, we discuss the possible pseudogap formation associated with the preformed pairs, which suppresses the DOS and hence leads to a change in quasiparticle scattering. We have measured the relaxation time *T*_1_ of ^77^Se NMR spectroscopy in FeSe single crystals (Supplementary Fig. 3) at different fields applied along the *c* axis. At 14.5 T close to the upper critical field, the temperature dependence of 1/*T*_1_*T*, which is dominated by the dynamical spin susceptibility *χ*(**q**) at the antiferromagnetic wave vector **q**=(*π*, *π*), can be fitted well by a Curie–Weiss law in a wide temperature range below *T*_s_ (Fig. 3a, inset). At low fields of 1 and 2 T, however, 1/*T*_1_*T*(*T*) shows a noticeable deviation from this fit (dashed line in Fig. 3a, inset), and the difference between the fit and the low-field data Δ(1/*T*_1_*T*) starts to grow at ∼T* (Fig. 3a, main panel). As the superconducting diamagnetism is an orbital effect that is dominated at *q*=0, the spin susceptibility *χ*(*π*, *π*) is not influenced by the orbital diamagnetism. Therefore, the observed deviation of 1/*T*_1_*T*(*T*) is a strong indication of a depletion of the DOS, providing spectroscopic evidence for the psedugap formation below ∼*T* *. The onset temperature and the field dependence of the non-linear contribution of 1/*T*_1_*T*(*T*) bear a certain similarity to the features of the diamagnetic susceptibility, pointing to the intimate relation between the pseudogap and preformed pairs in this system.

The pseudogap formation is further corroborated by the measurements of Hall (*R*_H_), Seebeck (*S*) and Nernst (*ν*) coefficients (Fig. 3b–d). The negative sign of the Hall and Seebeck data indicates that the transport properties are governed mainly by the electron band, which is consistent with the previous analysis of the electronic structure in the orthorhombic phase below *T*_s_ (ref. 16). Obviously, at *T* *∼20 K, all the coefficients show a minimum or maximum. Since the Hall effect is insensitive to superconducting fluctuations, the minimum of *R*_H_(∝(*σ*_h_−*σ*_e_)/(*σ*_h_+*σ*_e_)), where *σ*_e(h)_ is the conductivity of electrons (holes), suggests a change of the carrier mobility at ∼*T* *. The thermomagnetic Nernst coefficient consists of two contributions generated by different mechanisms: *ν*=*ν*_N_+*ν*_S_. The first term represents the contribution of normal quasiparticles. The second term, which is always positive, represents the contribution of fluctuations of either amplitude or phase of the superconducting order parameter. On approaching *T*_c_, *ν*_S_ is expected to diverge^30^. As shown in Fig. 3d, however, such a divergent behaviour is absent. This is because in the present very clean system, *ν*_N_ is much larger than *ν*_S_ (Supplementary Fig. 4a; Supplementary Note 2). Since *ν*_N_ and *S* are proportional to the energy derivatives of the Hall angle and conductivity at the Fermi level, respectively, 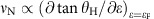 and 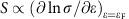 both sensitively detect the change of the energy dependence and/or anisotropy of the scattering time at the Fermi surface (see also Supplementary Fig. 4b,c for *ν*/*T*(*T*) and *S*/*T*(*T*)). Therefore, the temperature dependence of the three transport coefficients most likely implies a change in the quasiparticle excitations at *T* *, which is consistent with the pseudogap formation. We also note that anomalies at similar temperatures have been reported for the temperature dependence of the thermal expansion^13^ as well as of Young's modulus^29^. Recent scanning tunnelling spectroscopy data also suggest some suppression of the DOS at low energies in a similar temperature range^31^.

## Discussion

**Figure 4:**
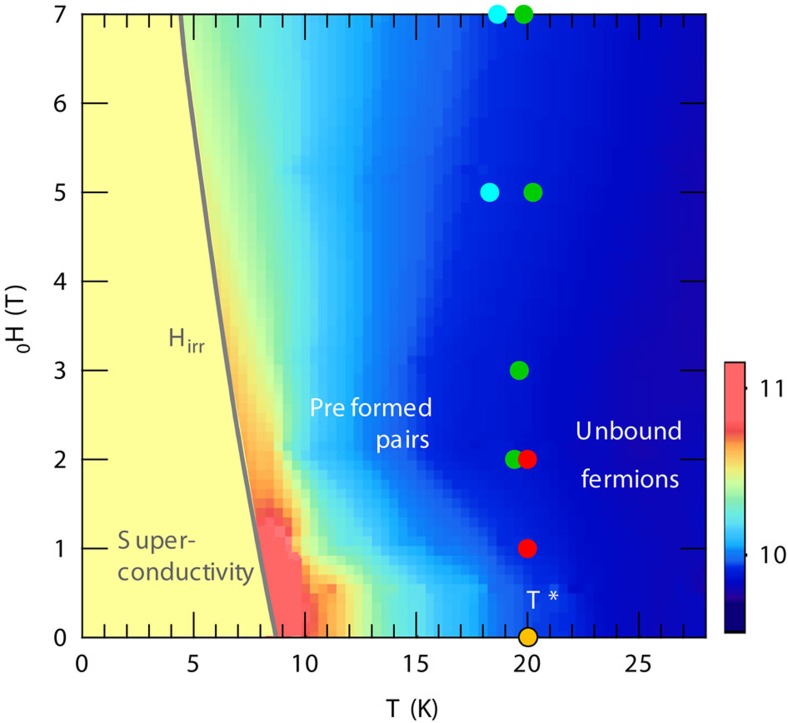
*H*–*T* phase diagram of FeSe for **H**||*c*. Solid line is the irreversibility line *H*_irr_(*T*) (ref. 14). The colour represents the magnitude of Δ*χ* (in 10^−5^, scale shown in the colour bar) from magnetic torque measurements (Fig. 2c). Preformed pair regime is determined by the minimum of d*ρ*_*xx*_(*H*)/d*T* (blue circles), the peak of Nernst coefficient *ν*_peak_ (green circles) and the onset of Δ(1/*T*_1_*T*) in the NMR measurements (red circles).

Figure 4 displays the schematic *H*–*T* phase diagram of FeSe for **H**||*c*. The fluctuation regime associated with preformed pairing is determined by the temperatures at which d*ρ*_*xx*_(*H*)/d*T* shows a minimum and *ν*(*H*) shows a peak (Supplementary Fig. 5a,b; Supplementary Note 3) in magnetic fields, as well as by the onset of Δ(1/*T*_1_*T*) (Fig. 3a). The diamagnetic signal, NMR relaxation rate and transport data consistently indicate that the preformed pair regime extends over a wide range of the phase diagram. The phase fluctuations dominate at low fields where the non-linear diamagnetic response is observed (Fig. 2d). This phase-fluctuation region continuously connects to the vortex liquid regime above the irreversibility field *H*_irr_, where a finite resistivity is observed with a broad superconducting transition (Fig. 1a).

Let us comment on the electronic specific heat, which is another thermodynamic quantity related to the DOS of quasiparticles. The specific heat *C* at comparatively high temperatures, however, is dominated by the phonon contribution ∝*T*^3^ (refs 29, 32), which makes it difficult to resolve the pseudogap anomaly. Also, the reduction of *C*/*T* may partly be cancelled with the increase by the strong superconducting fluctuations found in the present study. It should be also stressed that FeSe exhibits a semimetallic electronic structure with the compensation condition, that is, the electron and hole carrier densities should be the identical. Such a compensated situation of the electronic structure may alter significantly the chemical potential shift expected in the BEC theories for a single-band electronic structure. How the entropy in crossover semimetals behaves below *T* * is a fundamentally new problem, which deserves further theoretical studies.

Finally, we remark that the preformed Cooper pairs and pseudogap develop in the non-Fermi liquid state characterized by a linear-in-temperature resistivity, highlighting the highly unusual normal state of FeSe in the BCS–BEC crossover regime. The resistivity above *T* * can be fitted up to ∼50 K as *ρ*_*xx*_(*T*)=*ρ*_*xx*_(0)+*AT*^*α*^ with *α*=1.1−1.2, where the uncertainty arises from the fact that *ρ*_*xx*_(0) is unknown (Fig. 1b). Thus, the exponent *α* close to unity indicates a striking deviation from the Fermi liquid behaviour of *α*=2. This non-Fermi liquid behaviour in FeSe is reminiscent of the anomalous normal-state properties of high-*T*_c_ cuprate superconductors. The main difference between these systems and FeSe is the multiband nature of the latter^34^,^35^; the Fermi surface consists of compensating electron and hole pockets. The present observation of preformed pairs together with the breakdown of Fermi liquid theory in FeSe implies an inherent mechanism that brings about singular inelastic scattering properties of strongly interacting fermions in the BCS–BEC crossover.

## Methods

### Sample preparation and characterization

High-quality single crystals of tetragonal β-FeSe were grown by low-temperature vapour transport method at Karlsruhe Institute of Technology and Kyoto University^13^. As shown in Fig. 1b, taking *ρ*_*xx*_(*T*_c_^+^)≈10 *μ*Ω cm as an upper limit of the residual resistivity leads to the residual resistivity ratio (RRR)>40. The large RRR value, large magnetoresistance below *T*_s_, quantum oscillations at high fields^15^,^16^, a very sharp ^77^Se NMR line width^29^, and extremely low level of impurities and defects observed by scanning tunnelling microscope topographic images^14^,^33^, all demonstrate that the crystals used in the present study are very clean. The tetragonal structure is confirmed by single-crystal X-ray diffraction at room temperature. The tetragonal [100]_T_/[010]_T_ is along the square edges of the crystals, and below the structural transition, the orthorhombic [100]_O_/[010]_O_ along the diagonal direction.

### Magnetization and magnetic torque measurements

The magnetization was measured using a vibrating sample option (VSM) of the Physical Properties Measurement System by Quantum Design. Supplementary Figure 1 shows temperature dependence of the magnetization in a single crystal of FeSe for several different fields. We obtained the diamagnetic response in the magnetization, *M*_dia_, by shifting the curves to zero at 30 K, that is, by subtracting a constant representative of the normal-state magnetization ignoring the small paramagnetic Curie–Weiss contribution.

Magnetic torque is measured by using a micro-cantilever method^25^,^26^. As illustrated in the inset of Fig. 2b, a carefully selected tiny crystal of ideal tetragonal shape with 200 × 200 × 5 μm^3^ is mounted on to a piezo-resistive cantilever. The crystals contain orthorhombic domains with typical size of ∼5 μm below *T*_s_. Supplementary Figure 2a–f shows the magnetic torque *τ* measured in various fields, where the field orientation is varied within a plane including the *c* axis (*θ*=0,180°) and the field strength *H*=|**H**| is kept during the rotation. The torque curves at 0.5 and 1T (Supplementary Fig. 2a and b) are distorted at 8.5 K, which is expected in the superconducting state of anisotropic materials^36^ whereas those above 9 K are perfectly sinusoidal. 

### NMR measurements

^77^Se NMR measurements were performed on a collection of several oriented single crystals, and external fields (1, 2 and 14.5 T) are applied parallel to the *c* axis. Since ^77^Se has a nuclear spin *I*=1/2, and thus no electric quadrupole interactions, the resonance linewidth of the NMR spectra are very narrow with full width at half maximum of a couple of kHz (Supplementary Fig. 3). The nuclear spin-lattice relaxation rate 1/*T*_1_ is evaluated from the recovery curve *R*(*t*)=1−*m*(*t*)/*m*(∞) of the nuclear magnetization *m*(*t*), which is the nuclear magnetization at a time *t* after a saturation pulse. *R*(*t*) can be described by *R*(*t*)∝exp(−*t*/*T*_1_) with a unique *T*_1_ in the whole measured region, indicative of a homogeneous electronic state. In general, 1/*T*_1_ for *H*||*c* is related to the imaginary part of the dynamical magnetic susceptibility *χ*(**q**, *ω*) by the relation


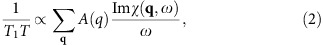


where *A*(*q*) is the transferred hyperfine coupling tensor along the *c* axis at the Se site and *ω*=*γ*_n_/*H* with *γ*_n_/(2*π*)=8.118 MHzT^−1^ is the NMR frequency. 1/*T*_1_*T* at the Se site is mainly governed by the magnetic fluctuations at the Fe sites, that is, particularly in FeSe, the short-lived stripe-antiferromagnetic correlations at **q**=(*π*, *π*) in the tetragonal notation. It should be noted that the superconducting diamagnetism is an orbital effect that is dominated at **q**=0 and thus it does not affect the dynamical spin susceptibility at **q**=(*π*, *π*).

### Thermoelectric measurements

The thermoelectric coefficients were measured by the standard d.c. method with one resistive heater, two Cernox thermometers and two lateral contacts (Fig. 3d, inset). The Seebeck signal *S* is the transverse electric field response *E*_*x*_ (||*x*), while the Nernst signal *N* is a longitudinal response *E*_*x*_ (||*x*) to a transeverse temperature gradient ∇_*x*_*T*(||*x*) in the presence of a magnetic field *H*_*z*_ (||*z*), that is, *S*≡*E*_*x*_/(−∇_*x*_*T*) and *N*≡*E*_*y*_/(−∇_*x*_*T*), respectively. The Nernst coefficient is defined as *ν*≡*N*/*μ*_0_*H*.

### Data availability

The data that support the findings of this study are available on request from the corresponding authors (T.S. or Y.M.).

## Additional information

**How to cite this article:** Kasahara, S. *et al*. Giant superconducting fluctuations in the compensated semimetal FeSe at the BCS–BEC crossover. *Nat. Commun.* 7:12843 doi: 10.1038/ncomms12843 (2016).

## Supplementary Material

Supplementary Information Supplementary Figures 1-5 and Supplementary Notes 1-3
