# Supplementary material for: Giant superconducting fluctuations in the compensated semimetal FeSe at the BCS–BEC crossover
Source: Nat Commun. 2016 Sep 30;7:12843. doi: 10.1038/ncomms12843 (PMC5056430; doi:10.1038/ncomms12843)
Supplement: Supplementary Information — Supplementary Figures 1-5 and Supplementary Notes 1-3 [file ncomms12843-s1.pdf]

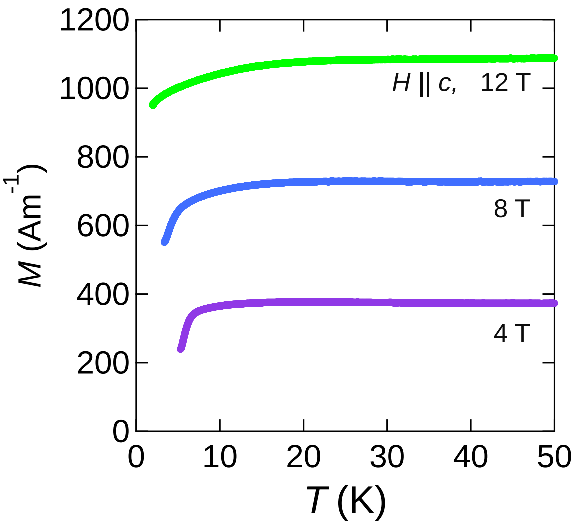

Supplementary Figure 1. Temperature dependence of magnetization for different fields applied along c axis.

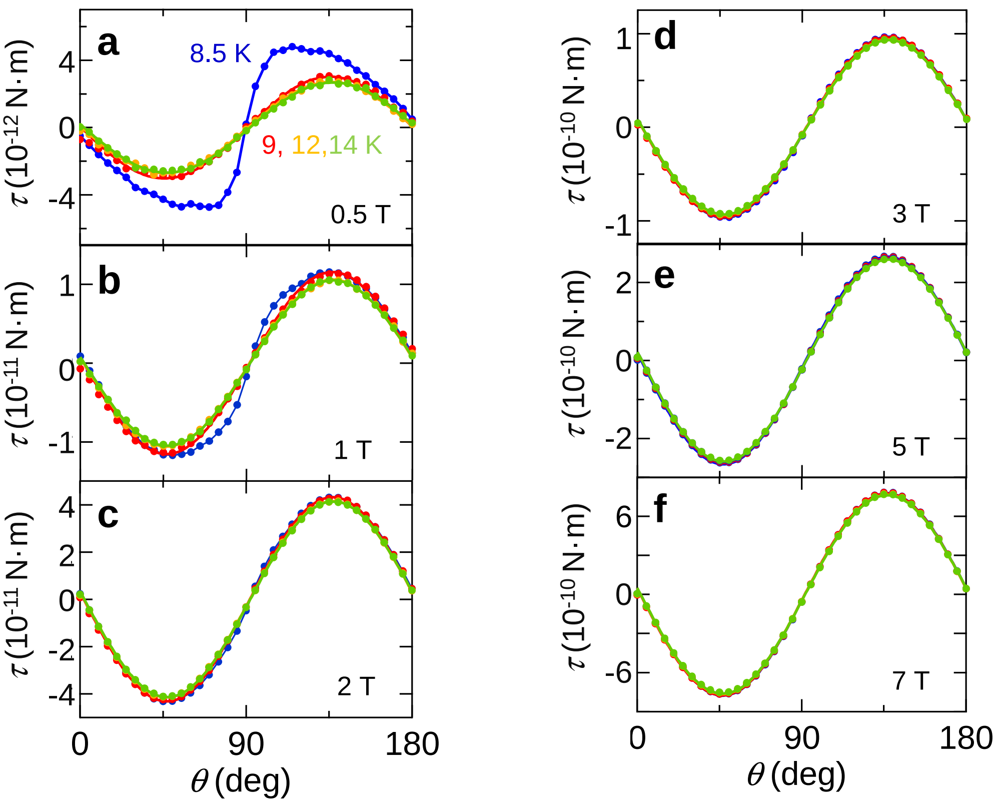

Supplementary Figure 2. Field angle dependence of magnetic torque for various fields and temperatures. θ is defined the angle between the field and the c axis.

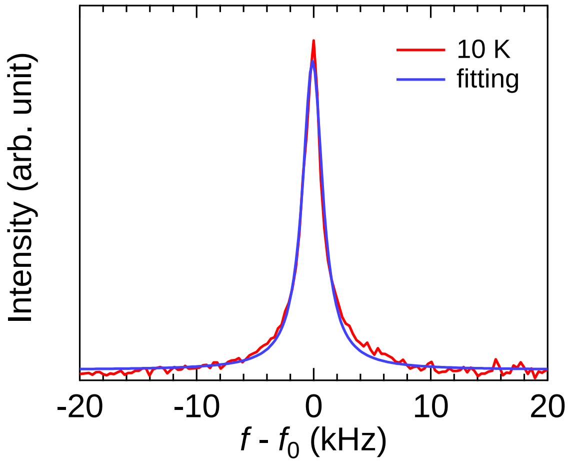

Supplementary Figure 3. **NMR spectrum at 10 K for $\mu_0 H = 1$ T applied along the c axis (red line).** Intensity is plotted against the relative frequency from the resonance frequency at f_0. A Lorentzian fit to the data (blue line) shows a narrow FWHM of ~ 2 kHz, indicating a homogeneous electronic state.

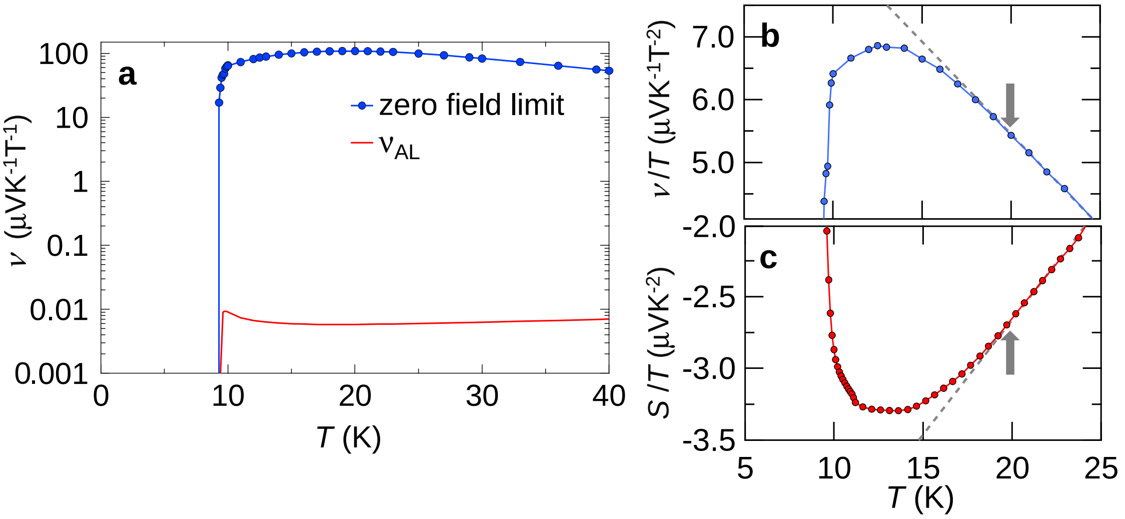

Supplementary Figure 4. **Temperature dependence of the thermoelectric coefficients.** **a**, Temperature dependence of the Nernst coefficient ν in the zero-field limit (blue circles) compared with the expected superconducting fluctuation contribution calculated from the AL theory Eq. (S1). **b**, ν/T as a function of temperature. **c**, Seebeck coefficient divided by temperature S/T as a function of temperature at zero field. Dashed lines are the linear fits at high temperatures. The arrows mark the psudogap temperature T^*.

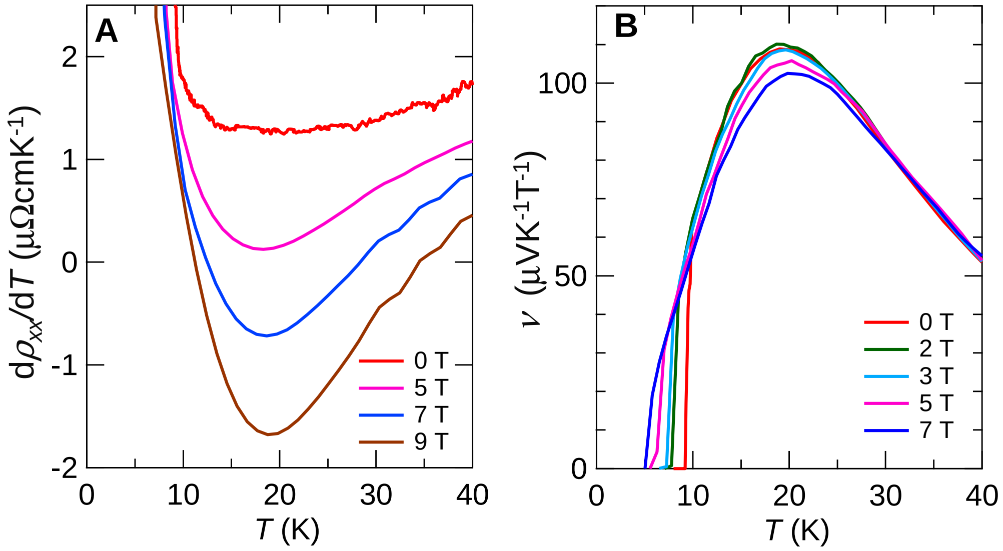

Supplementary Figure 5. **a**, Temperature derivative of the in-plane resistivity $d\rho_{xx}/dT$ and **b**, Nernst signal ν for different magnetic fields.

SUPPLEMENTARY NOTE 1. MAGNETIC TORQUE MEASUREMENTS

For the magnetic torque measurements, we selected crystals with no detectable ferromagnetic impurities, which show reversible sinusoidal torque curves with a hysteresis component of less than 0.01% of the total torque (Fig. 2a in the main text). At 0.5 and 1 T (Supplementary Fig. 2a and b), the curves are distorted at 8.5 K which is expected in the superconducting state of anisotropic materials [34], while those above 9 K are perfectly sinusoidal and are described well by $\tau(\theta, H, T) = A\sin(2\theta)$. All the torque curves above 2 T (Supplementary Figs. 2c-f) are perfectly sinusoidal in the normal state above T_c. The difference between the c-axis and ab-plane susceptibilities per unit volume, $\Delta\chi = \chi_c - \chi_{ab}$, is derived from Eq. (1) in the main text. We note that the torque signal above T_c is completely reversible without hysteresis, which excludes any ferromagnetic impurity as a source for the enhanced $|\Delta\chi|$ below T^*.

SUPPLEMENTARY NOTE 2. DOMINANT QUASIPARTICLE CONTRIBUTION IN NERNST SIGNAL

We estimate the Gaussian-type superconducting fluctuation contribution to the Nernst signal by $\nu_{AL} \approx \alpha_{xy}^{AL}\rho_{xx}/(\mu_0 H)$, where α_{xy}^{AL} is the Peltier coefficient [30]. In the Aslamasov-

Lakin (AL) theory, this coefficient is given by

$$\alpha_{xy}^{\rm AL}(T) = \frac{1}{12\pi}\frac{k_{\rm B}e}{\hbar}\frac{\tilde{\xi}_{ab}^2(T)}{\ell_H^2\tilde{\xi}_c(T)}, \tag{S1}$$

where $\tilde{\xi}_i(T) = \xi_i(0)/\sqrt{\ln(T/T_{\rm c})}$ ($i = ab$ or c) are the fluctuation coherence lengths parallel to the ab plane and c axis, and $\ell_{\rm H} = \sqrt{\hbar/2e\mu_0 H}$ is the magnetic length. The red line in Supplementary Fig. 4a represents the AL calculation for the superconducting fluctuation contribution $\nu_{\rm AL}(T)$ by using the resistivity data of the same sample and Eq. (S2). Just above $T_{\rm c}$, the measured data are almost four orders of magnitude larger than the AL estimate. Although the diamagnetic signal is one order of magnitude larger than the AL estimate (Fig. 2f), both the four-orders-of-magnitude difference and the absence of a diverging behaviour near $T_{\rm c}$ in $\nu(T)$ demonstrate that in our samples of FeSe the normal quasiparticle contribution dominates over the superconducting fluctuation contribution in the normal state.

As shown in Supplementary Figs. 4b and 4c, ν/T and S/T deviate from the high-temperature linear extrapolation below $T^* \sim 20$ K. At lower temperatures, ν/T and $|S|/T$ both decrease with decreasing T before superconductivity sets in, which is not expected in a Fermi-liquid metal where ν/T and S/T should become constant in the low-temperature limit.

SUPPLEMENTARY NOTE 3. FIELD DEPENDENCE OF IN-PLANE RESISTIVITY AND NERNST SIGNAL

In our clean crystals, a huge magnetoresistance is observed as shown in Fig. 1a of the main text. Due to the large value of $\omega_{\rm c}\tau$, where $\omega_{\rm c}$ is the cyclotron frequency and τ is the scattering time, ρ_{xx} under strong magnetic fields exhibits a semiconducting behaviour at low temperatures. In strong fields, superconducting fluctuations, or excess conductivity, are observed more clearly as they compete with the increase of ρ_{xx}. To see this, we plot the temperature derivative of the in-plane resistivity, $d\rho_{xx}/dT$, for several magnetic fields (Supplementary Fig. 5a). For each field, $d\rho_{xx}/dT$ exhibits a clear minimum at $T^* \sim 20$ K, indicating a slope change due to the emergence of superconducting fluctuations.

Supplementary Figure 5b shows the temperature dependence of the Nernst coefficient for different fields. Similar to $d\rho_{xx}/dT$, $\nu(T)$ at each field exhibits a clear peak at $T^* \sim 20$ K,

suggesting a change of the energy dependence of the scattering time at the Fermi level. We note that the anomalies in $d\rho_{xx}/dT$ and $\nu(T)$, as well $M_{\rm dia}$ are less sensitive to magnetic field, suggesting that they represent the amplitude fluctuations, while the non-linear behaviour in magnetic torque is readily suppressed by a strong magnetic field. This suggests that the non-linear diamagnetic response in torque is directly related to the phase fluctuations arising from the mode coupling of superconducting fluctuations.
